# Pregnant and postpartum women’s experiences of weight stigma in healthcare

**DOI:** 10.1186/s12884-020-03202-5

**Published:** 2020-08-27

**Authors:** Angela C. Incollingo Rodriguez, Stephanie M. Smieszek, Kathryn E. Nippert, A. Janet Tomiyama

**Affiliations:** 1 Department of Social Science and Policy Studies, Worcester Polytechnic Institute, 100 Institute Road, Worcester, MA 01609 USA; 2 Department of Psychology, University of California, 502 Portola Plaza, Los Angeles, CA 90095 USA

**Keywords:** Maternal health, Maternal obesity, OBGYN, Prenatal healthcare, Postpartum healthcare, Weight stigma

## Abstract

**Background:**

Weight stigma is a societal phenomenon that is very prevalent in healthcare, precipitating poor patient-provider relationships, discontinuity of care, and delayed cancer screening. Little research, though, has investigated weight stigma in prenatal and postpartum healthcare. To address this gap, this study examined the prevalence and frequency of weight-stigmatizing experiences in prenatal and postpartum healthcare.

**Methods:**

501 pregnant and postpartum women responded to an online survey where they reported whether they had experienced weight stigma in prenatal or postpartum healthcare and, if so, how frequently. Participants also responded to questions about how providers had treated them regarding their weight and their reactions to these experiences. A subset of participants (*n* = 80) also provided examples of their experiences, and these were subjected to a thematic analysis and coded for overarching themes.

**Results:**

Nearly 1 in 5 women (*n* = 92) reported experiencing weight stigma in healthcare settings. Percentages differed by BMI, with 28.4% of participants with pre-pregnancy obesity endorsing healthcare providers as a source of weight stigma. Experiences occurred between “less than once a month” and “a few times a month.” Obstetricians were the most commonly-reported source (33.8%), followed by nurses (11.3%). Participants reported feeling judged, shamed, and guilty because of their weight during healthcare visits. Additionally, 37 participants (7.7%) reported having changed providers because of treatment regarding their weight. Many also reported that they expected to feel or had felt uncomfortable seeking help with breastfeeding from a healthcare professional. Finally, thematic analysis of the open-ended examples identified four key themes: (1) negative attitudes and unkind or disrespectful treatment from providers; (2) evaluative comments about their weight; (3) healthcare providers focusing on their high-risk status and potential negative consequences (often when birth outcomes were ultimately healthy); and (4) inappropriate or demeaning comments.

**Conclusions:**

Weight stigma may be a common experience in pregnancy and postpartum healthcare. Providers need additional training to avoid stigmatizing their patients and inadvertently undermining patient-provider relationships, quality of care, and health outcomes.

## Background

Regular healthcare visits are important and common in the typical pregnancy experience in advanced economies. During increasingly frequent check-ups, pregnant women interact with staff, nurses, sonographers, and obstetrician/gynecologists (OBGYNs). Although these interactions serve the larger goal of promoting and protecting the health of the mother and child, healthcare settings can be a context where patients experience weight stigma – bias or discrimination based on one’s weight [1] – which can undermine this very goal [2].

Healthcare providers are understandably concerned with obesity as it is associated with several adverse health outcomes [3]. However, hyper-focusing on obesity can come with the unintended consequence of enacting weight stigma as well. Indeed, research shows that doctors, nurses, and dieticians consistently endorse negative stereotypes about heavier patients. In particular, providers typically blame patients for their weight [4], and obesity researchers and healthcare professionals specializing in obesity demonstrate implicit and explicit anti-fat bias, with the latter having increased over time [5]. This trend is ongoing as future healthcare provider cohorts display such bias as well. One study found that among medical students in the United States, 74% of them harbored implicit weight bias and 67% reported explicit weight bias [6]. Furthermore, these negative attitudes toward individuals with overweight and obesity were stronger than bias toward racial minorities, sexual minorities, and individuals of low socioeconomic status (SES).

The unfavorable consequences of such social stigma are numerous. Of particular relevance here, weight stigma in healthcare settings creates a barrier for treatment goals and subsequent outcomes. Namely, experiencing weight stigma from a provider hinders patient-provider communication and relationships, leading to reduced quality of care, non-adherence, and worse treatment outcomes [2, 7]. Such experiences also promote “doctor shopping” (i.e., frequently changing healthcare providers), which interrupts continuity of healthcare. For instance, in a sample of 20,000 patients, those with obesity had 52% higher odds of doctor shopping compared to patients with a normal weight Body Mass Index (BMI). Additionally, patients with overweight and obesity who engaged in doctor shopping had higher rates of emergency department visits by 83–85% [8].

Weight stigma also pervades women’s health specifically and can undermine women’s care and goals. For instance, a study of 149 racially diverse and low-SES women with obesity found that the more frequently a woman experienced weight stigma in healthcare settings, the greater the odds that she would have a negative perception of her physician’s empathy [9]. Objective BMI, though, was not associated with perceptions of care. Another study of nearly 500 White and Black women found that women with obesity put off important cancer screening tests such as pelvic exams, Pap tests, and mammograms because of weight-related barriers [10]. Many of these reported barriers involved weight stigma, such as being treated disrespectfully, encountering negative attitudes from providers, feeling embarrassed while being weighed, and receiving unsolicited recommendations to lose weight.

Compared to the broader weight stigma literature, a smaller body of prior research suggests that pregnant and postpartum women also experience weight stigma in healthcare settings. For instance, a qualitative interview study of six pregnant and ten postpartum women with obesity in the United States reported that most had had at least one negative encounter throughout their perinatal care experience [11]. Moreover, the majority of these women also reported experiencing depersonalized care that set a negative tone for their pregnancy and subsequent pregnancies. In a study of 627 Australian pregnant women, BMI was associated with negative experiences in healthcare settings [12]. In this same study, 248 Australian healthcare providers reported perceiving heavier mothers as having poor self-management behaviors, and they reported having negative attitudes toward caring for these women. A study of 19 women with obesity in the United Kingdom revealed they often felt humiliated during healthcare visits. Moreover, healthcare providers over-medicalized these pregnancies, making assumptions about health and abilities based on weight [13]. Lastly, 24 heavy Canadian women reported experiencing “mother-blame” where healthcare providers characterized weight-related fetal risk as indicative of unfitness for motherhood [14, 15]. In this study, many women also experienced alarming preconception practices, including providers refusing to remove implanted contraceptives such as an intrauterine device.

Weight stigma also paradoxically undermines the very goal of treating obesity – weight loss. Weight stigma, regardless of BMI, promotes obesogenic behaviors, like increased eating and reduced exercise [16], and weight gain over time [17, 18]. With the rates of pre-pregnancy obesity at 22% in the United States [19] and excess gestational weight gain at 47% [20], addressing weight and weight gain in prenatal and postpartum care is essential. Recent evidence, though, shows that experiencing pregnancy-related weight stigma is associated with unhealthy eating, gestational weight gain, and postpartum weight retention [21, 22]. Therefore, stigmatizing patients may ironically hinder attempts to curtail gestational weight gain.

To improve prenatal and postpartum healthcare and to avoid unintended negative consequences of weight stigma, detailed evidence of weight stigma in healthcare settings is needed. The present study surveyed pregnant and postpartum women using both questionnaire and open-response formats. Quantitative methods were used to investigate the reported prevalence, frequency, and sources of weight stigma in pregnancy and postpartum healthcare in the United States. We hypothesized that heavier women would report weight-stigmatizing encounters in healthcare at greater rates and generally more negative overall perceptions of their healthcare experiences. Qualitative thematic analysis techniques were used to characterize participants’ personal narrative examples of experiences and identify recurring themes.

## Methods

### Participants

Recruitment targeted women 18 years and older who were at least 13 weeks pregnant or less than one year postpartum. Exclusion criteria were living outside the United States and having a multiple gestation pregnancy. The final sample consisted of 501 women (28.5%, *n* = 143 pregnant; 71.5%, *n* = 358 postpartum). These women resided in 48 states around the United States, with California being the most represented state (16.8% residence).

Some background information on the United States pre- and postnatal guidelines may be useful in contextualizing this sample: The American Congress of Obstetrics and Gynecology and the American Academy of Pediatrics recommend the following prenatal healthcare visit schedule: every four weeks until 28 weeks of gestation; every 2 weeks from 28 weeks until 36 weeks gestations; and weekly from there until delivery. Healthy births without complications should be followed by a visit six weeks after delivery [23]. Data from every registered birth across all 50 states in the United States in 2016 revealed that more than 75% of women received adequate prenatal care, defined by completing more than 80% of recommended visits [24]. This report also documented that more than two thirds of women (77.1%) initiate care in the first trimester (i.e., less than 13 weeks gestation), and most women pay for their prenatal care via Medicaid, private insurance, or self-pay. Based on United States national guidelines and the above data, these women were currently or had very recently received prenatal and/or postpartum healthcare.

### Procedure

The university’s Institutional Review Board approved all procedures. Data were collected between August and November of 2017. Recruitment activities included posting flyers in healthcare offices, cafés, childcare facilities, and baby retail locations in the wider Los Angeles, California area. These flyers included pull-off tabs with a link to Qualtrics. Qualtrics is an online platform with secure software using secure data centers that adhere to the Health Information Technology for Economic and Clinical Health Act, HIPAA, 21 CFR 11 (and other regulation provisions relevant here), and EU data privacy directives. Further information on data security is available at https://www.qualtrics.com/security-statement/. Participants were also recruited through online forums for expectant and new mothers, such as on Facebook, Yahoo!, and Instagram. An electronic version of the flyer was posted on these forums along with the Qualtrics link for participation. Participants were able to read the informed consent electronically and check a box indicating their agreement to participate. Those who agreed then completed the web-based survey, consisting of questionnaires and open-response questions. Participants could skip any question they were uncomfortable answering and still remain in the study. All data were collected anonymously, but participants had the option of providing an email address for a raffle of five $100 prizes. Email addresses were examined to ensure no one had participated twice. Data were stripped of this information prior to analyses. Responses were stored in Qualtrics until data collection was completed, at which point data were removed from the online platform and stored on a secure server.

### Measures

Measures were adapted from the weight stigma and pregnancy/postpartum literatures. New measures were also designed for this study based on semi-structured pilot interviews that were conducted with six pregnant and postpartum women.

#### Weight stigma in healthcare

Participants were asked if they had ever been treated differently or made to feel bad or uncomfortable because of their weight since becoming pregnant. This characterization of a weight-stigmatizing experience was adapted from previous work [25, 26]. Participants selected all the following sources that applied: work, immediate family, extended family, friends, church, partner/spouse, healthcare providers, strangers, media, other mothers, society, or other. For each source endorsed, participants were asked to provide an example of their experiences in open-response format. The prompt read as follows: “So that we can fully understand what happened, for each of the people or situations you selected above, please provide an example of one of these experiences. Make sure to describe who/what made you feel bad or treated you differently and how it happened.” A total of 92 participants endorsed healthcare provider as a source of weight stigma, and of those, 80 provided an example.

Lastly, they reported the frequency of weight-stigmatizing experiences for each source. Response options ranged from “less than once a month” to “3 or more times daily.”

#### Healthcare experiences

All participants received the following questions designed based on pilot interviews:

Participants characterized their overall experiences with prenatal, labor and delivery, and postpartum healthcare, separately, selecting from a five-point scale from one “very negative” to five “very positive.” A score of three represented the “neutral” point on the scale.

Participants were asked if they had ever felt any of the following during healthcare interactions because of their weight: valued, important, accepted, judged, shamed or ashamed, guilty, less worthy, invisible, unimportant, disrespected, negatively compared to other patients, disliked, or as though the provider thought they were stupid or unintelligent. Participants selected all providers who had made them feel this way from the following: physician, nurse or nurse assistant, physician’s assistant, midwife or doula, ultrasound technician, office staff, fill in the blank. They were also asked in what type of practice the weight stigma had occurred: hospital-affiliated OBGYN, private practice OBGYN, birthing center, public clinic, urgent care, hospital emergency room or other specialist, fill in the blank.

Participants reported if they had ever changed prenatal healthcare providers because of how the provider had treated them regarding their weight. Participants also indicated whether they felt that too little, the right amount, or too much attention was paid to their weight or weight gain. Lastly, participants were asked if they had felt they could not trust their doctor or had to advocate for or stand up for themselves because the doctor focused too much on weight.

#### Breastfeeding

Participants reported whether they believed they would feel uncomfortable or had ever felt uncomfortable seeking help with breastfeeding from a healthcare professional. If affirmative, they were asked if this was because of their weight.

#### Pregnancy and postpartum status

Participants reported on parity dichotomously (primiparous or multiparous) and weeks of gestation or months postpartum.

#### Weight information

Participants reported height in inches and pre-pregnancy weight in pounds. Pre-pregnancy BMI was calculated as weight (lb)/[height (in)]^2^*703 and categorized according to the standard cutoffs for “underweight” (< 18.5), “normal weight” (18.5–24.9), “overweight” (25.0–29.9), and “obese” (≥ 30.0).

#### Demographic information

Participants reported age, highest completed education, race/ethnicity, household size, zip code, and household income. Household size and income were used to calculate household income per capita and poverty status for 2017 ($12,060 plus $4180 for each additional person).

Exhaustive information on methods is available elsewhere [21, 27]. See supplementary material for the original versions of the weight stigma, healthcare experiences, and breastfeeding questionnaires developed for this study.

### Data analytic plan

Descriptive statistics evaluated frequency of weight stigma from healthcare providers and characterized ratings of healthcare experiences. Correlation analyses and one-way ANOVAs tested the role of continuous BMI in these relationships. A chi-square analysis examined differences in endorsement rates by BMI category. Open-ended responses were subjected to thematic analysis using Braun and Clark’s six-step guide [28]. Step 1: The first and second authors familiarized themselves with the responses provided by participants. Step 2: The second author developed a coding scheme to capture recurrent themes that emerged during step 1. The second and third authors independently coded all responses based on this scheme. The first and third authors resolved any discrepancies in these two waves of coding. Step 3: The third author reviewed the coding results for overarching themes across the responses. Steps 4–5: The first author reviewed, defined and named these themes and aggregated key examples of each. Step 6: The first, third, and fourth authors together produced the coding report and overarching themes.

## Results

### Sample characteristics

The full sample had an average pre-pregnancy BMI of 33.66 ± 11.19; 17.2% were “overweight” and 54.1% “obese.” The sample was primarily White (67.3%) and on average 28.31 years old. The majority (63.2%) had completed post-secondary education. About half (50.5%) lived above 200% of poverty.

The subsample who endorsed healthcare providers as a source of weight stigma (*n* = 92) did not significantly differ from the larger sample on any demographics (all *p*’s > .22). This subsample had a higher average pre-pregnancy BMI (*M* = 42.67 ± 10.29, range: 17.72–71.57) than the group that did not endorse healthcare providers as a source (*M* = 31.65 ± 10.40, range: 15.11–74.39), *F* (1, 499) = 84.63, *p* < .001. However, both groups had average pre-pregnancy BMIs in the “obese” range. Proportionate to the full sample, 31.5% (*n* = 29) were pregnant and 68.5% (*n* = 63) postpartum.

See Table 1 for characteristics of the full study sample as well as the subsample who endorsed experiencing weight stigma from healthcare providers. More detailed demographics are available elsewhere [21, 27].

**Table 1:** Characteristics of the Full Sample and Subsample Endorsing Healthcare as a Source of Stigma

Variable	Overall (*N* = 501)	Subsample Endorsing Healthcare (*n* = 92)
Status		
Pregnant	28.5%	31.5%
Postpartum	71.5%	68.5%
Age (years)	28.31 (5.15)	28.78 (4.83)
Race/Ethnicity		
White	67.3%	75%
Black	2.8%	4.3%
Latina	10.2%	6.5%
Asian/Pacific Islander	2.8%	–
Other or multiracial	2.2%	2.2%
Not reported	14.8	12%
Income per capita (in thousands of dollars)	21.94 (19.53)	24.36 (19.82)
Pregnant with/delivered first child	52.9%	48.3%
Pre-pregnancy BMI	33.66 (11.19)	42.67 (10.29)
Pre-pregnancy BMI categories		
“Underweight”	2.6%	1.1%
“Normal weight”	26.1%	4.3%
“Overweight”	17.2%	10.9%
“Obese”	54.1%	83.7%

*Note*. Numbers in parentheses are standard deviations

### Endorsement and frequency

The percent of women from the full sample who endorsed healthcare providers as a source of weight sigma differed significantly by pre-pregnancy BMI category, *χ*^2^(2) = 20.36, *p* < .001. In general, a greater proportion of women with pre-pregnancy obesity endorsed healthcare providers compared to those who were “normal weight” or “overweight”. In the full sample, there were 144 participants with a normal weight pre-pregnancy BMI. Of those, five (3.5%) endorsed healthcare providers as a source of weight stigma. There were 86 participants with an overweight pre-pregnancy BMI in the full sample, and of those 10 (11.6%) endorsed healthcare providers. Finally, there were 271 participants in the full sample with an obese pre-pregnancy BMI, and of those, 77 (28.4%) endorsed healthcare providers. The average frequency of experiences was 1.54 ± 0.58, which falls between “less than once a month” and “a few times a month.” The correlation between frequency of experiences and pre-pregnancy BMI was non-significant, *r* (92) = .16, *p* = .126.

### Healthcare-specific questions

In general, participants described their overall experiences in healthcare as positive. The average rating for prenatal healthcare was 4.22 ± 1.00, which falls just above “somewhat positive” on the response scale. For labor and delivery care, the average rating was similarly 4.23 ± 1.11. For postpartum care, the average was 3.82 ± 1.20, which falls between “neutral” and “somewhat positive.” Pre-pregnancy BMI was not significantly correlated with these ratings (all *p*’s > .21). Further, even participants who endorsed healthcare providers as a source of stigma had overall ratings in the positive range: 3.55 ± 1.09 for prenatal; 3.62 ± 1.32 for labor and delivery; and 3.31 ± 1.23 for postpartum healthcare.

Many participants also endorsed having felt positive feelings – valued (28.3%), important (26.5%), and accepted (54.9%) – while interacting with healthcare providers. However, participants also endorsed having felt negative feeling such as judged (23.4%) and shamed (15.8%) because of their weight. Participants who endorsed healthcare providers as source of weight stigma consistently endorsed the positive emotions at lower rates and the negative emotions at higher rates. See Table 2 for the percent of participants endorsing each feeling experienced in healthcare settings in the overall sample and in the subsample that endorsed healthcare providers as a source of weight stigma.

**Table 2:** Feelings Experienced while Interacting with Healthcare Providers

Feeling	Overall (*N* = 501)	Subsample Endorsing Healthcare (*n* = 92)
Valued	28.3%	13%
Important	26.5%	10.9%
Accepted	54.9%	15.2%
Judged	23.4%	71.7%
Shamed	15.8%	51.1%
Guilty	14.4%	43.5%
Less Worthy	12.8%	44.6%
Invisible	3.2%	13%
Unimportant	11%	28.3%
Disrespected	9.6%	32.6%
Negatively compared to others	11.6%	35.9%
Disliked by the provider	9.4%	37%
As if the provider thought they were stupid or unintelligent	11.4%	38%

Additionally, among all 501 participants, 7.7% reported changing pregnancy or postpartum healthcare providers because of weight-related treatment. Additionally, 12.3% reported that too little attention was paid to weight or weight gain, 76.1% the right amount, and 11.6% too much. 11.2% reported feeling they could not trust their doctor or that they had to advocate for themselves because the doctor focused too much on weight.

Among pregnant women, 21.5% (*n* = 28) said they expected they would feel uncomfortable seeking help with breastfeeding from a healthcare professional. Of those, 48.1% (*n* = 13) said this was because of their weight. Among postpartum women, 25.7% (*n* = 73) said they had felt uncomfortable seeking help with breastfeeding. Of those, 41.1% (*n* = 30) said this was because of their weight.

### Relationships with BMI

The above-reported relationships consistently differed based on pre-pregnancy BMI. For instance, participants who reported having changed their provider had a significantly higher pre-pregnancy BMI (*M* = 42.79 ± 10.89) than those who did not (*M* = 32.92 ± 10.91), *F* (1, 478) = 28.02, *p* < .001.

There was a significant difference in pre-pregnancy BMI among women who reported that too little (*M* = 31.29 ± 9.19), the right amount (*M* = 32.97 ± 11.07), and too much (*M* = 40.69 ± 11.58) attention was paid to their weight, *F* (2, 478) = 13.73, *p* < .001. Post-hoc analyses revealed those reporting too much attention had a significantly higher pre-pregnancy BMI than others.

Those reporting that they could not trust their provider because of weight-related treatment also had significantly higher pre-pregnancy BMIs (*M* = 40.67 ± 10.64) than those who did not (*M* = 32.78 ± 10.97), *F* (1, 479) = 24.95, *p* < .001.

Pregnant participants who expected that they would feel uncomfortable seeking help with breastfeeding had a marginally significantly higher pre-pregnancy BMI (*M* = 40.28 ± 11.84) than those who did not (*M* = 34.20 ± 12.46), *F* (1, 128) = 3.73, *p* < .056. For postpartum participants, those who had felt uncomfortable seeking help with breastfeeding had significantly higher pre-pregnancy BMIs (*M* = 36.01 ± 11.76) than those who had not (*M* = 32.28 ± 10.20), *F* (1, 282) = 6.68, *p* = .010.

### Thematic analysis

In the subset of 92 women who had endorsed healthcare providers as a source, 80 provided an example of their experiences. After familiarization with the responses overall, a coding scheme was developed to capture recurring themes, and each of the 80 examples were evaluated for the presence of each of these codes. The codes included the type of stigma participants had experienced (e.g., verbal, nonverbal); whether their treatment was positive or negative; the types of providers; the providers’ gender; whether the providers discussed health consequences due to maternal weight; whether the providers made assumptions about participants’ health or health behaviors; whether the participants had been “threatened” with a C-section; and whether they had been referred to a specialist.

Based on this coding process, the following overarching themes were identified (see Table 3 for more examples):

**Table 3:** Overarching Themes and Examples from Thematic Analysis

Theme	Examples
Negative attitudes and unkind/disrespectful treatment	• At almost every visit, I had at least one negative comment or a person who expressed frustration with my weight … • … speaking to me like I was a complete idiot. He kept saying “we are all VERY VERY worried about you.” In a very demeaning way. • I don’t want to be chastised like a child.
Evaluative comments about weight	• Doctor constantly telling me I’m too heavy. I need to be doing something about it. • “How did you let yourself get so big?” • Doctors always talk negatively about my weight. • Saying I’m too big and need to lose weight.
High-risk status and negative outcomes … … despite healthy outcomes	• I was told I’m automatically high risk, that I was almost certain to have [gestational diabetes], that I would need a C-section, and people of my size generally do not have vaginal births like I wanted. • One doctor told me that … I would probably get preeclampsia, gestational diabetes, and that the surgeon may not even want to work with me. • I was told that I would probably be high risk with hypertension and/or preeclampsia and/or [gestational diabetes] because of my weight. • Nurses always assumed that with my weight there would be risk or [I would have preeclampsia]. Turns out they were wrong. I was healthy and my son was healthy even though I’m plus size. • OBGYN acted extremely surprised at my appointments when my blood pressure was normal and I passed all the gestational diabetes tests as if there was no way I could have a complication free pregnancy at my weight. • There’s nothing wrong with me. I’m having a textbook pregnancy other than the fact that I’m obese.
Inappropriate comments	• “Do you want a vaginal delivery or a donut. Your vagina gains weight, too.” • One doctor told me I was terrible for getting pregnant at my weight, that I was setting up my baby to fail … I was in tears, and he told me I was being too sensitive”

*Note*. Typos have been edited

(a) Negative attitudes and unkind or disrespectful treatment, such as “my first doctor was very rude about my weight.”

(b) Evaluative comments about weight, such as “[you’re] too heavy.”

(c) An intense focus on high-risk status and potential negative outcomes based solely on the woman’s weight. One woman reported being told at eight weeks pregnant, “you’re too big and you’ll need to be induced.” Often, this preempted worry and prediction of negative consequences occurred in spite of other healthy test results and, ultimately, healthy birth outcomes. For instance, one woman reported, “The lady made me feel like I couldn’t have a healthy pregnancy. Well I did! No GD and a vaginal birth no meds!”

(d) Inappropriate comments. These included name-calling and pejorative comparisons such as being called, “sugar-holic.” Providers also made insensitive, and unfounded, comments about participants’ mothering ability or the future mother-child relationship, such as “[your] weight gain will embarrass [your] child if [you] do not get fit...”

## Discussion

The present study revealed that many expecting and new mothers in the United States report experiencing weight stigma from healthcare providers, in particular from physicians and nurses. A portion indicated that this treatment led them to mistrust their doctors, and for some, to even change providers. Additionally, thematic analysis revealed that many women experienced some form of negative treatment, which had multiple manifestations from impolite comments to disrespectful belittling.

Importantly, the average frequency of weight-stigmatizing experiences fell between “less than once a month” and “a few times a month.” This frequency is consistent with the typical occurrence of healthcare visits during pregnancy in the United States, indicating women may experience weight stigma regularly and frequently in healthcare. This may have clinical implications considering the abovementioned literature linking weight stigma to poor patient-provider relationships, healthcare avoidance, and doctor shopping. In light of known higher health risk for heavier mothers, it is critical that these women, especially, maintain consistent healthcare.

Unduly focusing on potential negative consequences for the mother’s and/or child’s health, a common theme reported here, may also be counterproductive. In fact, research shows that higher medical risk and perceived risk of complications are themselves risk factors for pregnancy anxiety [29]. Experiencing pregnancy anxiety – which stems from worries about the baby’s health, childbirth, and motherhood – increases risk for impaired fetal neurodevelopment, preterm birth, and child cognitive and motor deficits [29–31]. Reminding a woman in a stigmatizing manner that her weight will make childbirth harder, harm her baby, or impair the mother-child relationship could in fact activate all the key components of pregnancy anxiety and, in turn, increase risk. Doctors may be concerned about potential consequences of maternal weight, but increasing pregnancy-related anxiety can itself be deleterious as well.

On top of this, stigmatizing comments, while perhaps well-intentioned, are simply not helpful in reducing and preventing maternal obesity. Weight stigma experienced by pregnant and postpartum women is associated with more gestational weight gain and postpartum weight retention [21, 22]. Therefore, not only are weight-stigmatizing comments unkind and distressing, they are not particularly productive either. These comments may even compel women to engage in potentially unhealthy and dangerous behaviors. For instance, one woman reported, “I was told that I shouldn’t gain any weight with my pregnancy. I took it as a challenge. I didn’t gain any.” Insufficient weight gain can put the child at risk as it is associated with preterm birth and low birth weight [32, 33], and weight stigma itself is associated with maladaptive dieting behaviors in pregnancy and postpartum [21]. This issue is a complicated one though, as some research suggests that specifically for women with obesity, weight loss during pregnancy might lower risk for certain adverse outcomes such as pre-eclampsia, excessive bleeding, and fetal distress but increase risk for having a small-for-gestational-age infant [34]. Providers therefore must take an individualized approach to each patient’s weight gain. Additionally, they should bear in mind that the potential negative downstream consequences of stigmatizing mothers for their weight – from discontinuity of care to undermining health – may run the risk of not being balanced by promoting healthy weight or behaviors. Therefore, compassionate care, free from stigma, would promote the goals of both healthcare provider and patient.

These findings also suggest, albeit preliminarily, that weight stigma may discourage women from breastfeeding or seeking a provider’s help with breastfeeding. This may partially account for the relationship between obesity and decreased breastfeeding. In previous research on White women, those with obese versus normal weight BMIs were less likely to initiate breastfeeding and more likely to stop within six months [35]. A study of postpartum women documented that pre-pregnancy BMI significantly predicted never breastfeeding among non-Hispanic White and Black women [36]. Moreover, even when women believe breastfeeding is best, many choose to bottle feed because of negative expectations and attitudes toward breastfeeding that they develop during pregnancy [37]. Therefore, understanding the expectations that heavy pregnant women have towards breastfeeding may help reverse this trend. In this case, heavy White women, a characterization that applies to the majority of this sample, may have negative expectations about breastfeeding and likely have a heightened need for breastfeeding support. Yet they may not seek that support.

Compounding this issue, weight stigma is associated with postpartum depression symptoms regardless of BMI [21, 22]. Postpartum depression is also a risk factor for breastfeeding difficulty and early cessation [38]. Thus, weight stigma may filter through multiple avenues to hinder breastfeeding efforts and shorten duration. This is a public health concern given the known consequences of not breastfeeding for maternal-child morbidity and mortality and added financial healthcare burden of not breastfeeding [39]. Further research should investigate weight stigma’s impact on breastfeeding in greater detail.

The interpretation of these findings must account for this sample being primarily White and of higher SES. Additionally, a large portion of the sample came from California. To achieve an inclusive and generalizable understanding of this issue, future research should investigate weight stigma in more representative samples. This is especially necessary in healthcare settings serving low-income and racial/ethnic minority mothers. Additionally, pre-pregnancy BMI was based on self-reported weight. However, self-report error for pre-pregnancy weight is typically low [40], and pre-pregnancy BMI categorization from self-reported pre-pregnancy weight is usually correct [41].

## Conclusions

In sum, this work suggests that weight stigma may be problematic in maternal healthcare. In light of the known consequences of weight stigma, especially in the context of pregnancy and postpartum, providers need new approaches for discussing weight with heavy mothers. This is a major challenge for healthcare providers, especially considering that research shows that providers tend to lack training and skills for communicating weight gain guidelines [42]. Providers would likely benefit from further training to not only address these guidelines, but to do so in a non-stigmatizing fashion. While it may be necessary to inform women of risks that can accompany their weight or weight gain, doing so in a rude, belittling, or demeaning fashion must be eliminated. Further research on the representative prevalence of weight stigma in prenatal and postpartum healthcare and how to mitigate it is therefore needed. This way, providers can fulfill the true intent of this healthcare without potentially and paradoxically precipitating distress, ill health including weight gain, and discontinuity of care.

## Supplementary information

**Additional file 1.** Original Questionnaires. Original versions of questionnaires developed for this study: Sources and Frequency of Weight Stigma; Specific Questions about Healthcare; and Specific Questions about Breastfeeding

## Data Availability

All data generated or analyzed during this study are available from the corresponding author on reasonable request.

## Abbreviations

BMI: Body Mass Index
OBGYN: Obstetrician/gynecologist
SES: Socioeconomic status
