# Supplementary material for: Pregnant and postpartum women’s experiences of weight stigma in healthcare
Source: BMC Pregnancy Childbirth. 2020 Aug 27;20:499. doi: 10.1186/s12884-020-03202-5 (PMC7457255; doi:10.1186/s12884-020-03202-5)
Supplement: Supplementary file 1 — Additional file 1. Original Questionnaires. Original versions of questionnaires developed for this study: Sources and Frequency of Weight Stigma; Specific Questions about Healthcare; and Specific Questions about Breastfeeding [file 12884_2020_3202_MOESM1_ESM.docx]

**Original Questionnaires**

**Sources and Frequency of Weight Stigma**

1. Some people are made to feel good or bad about how they look. **Since becoming pregnant**, have you ever been treated differently because of your weight or has something or someone made you feel bad or uncomfortable because of your weight? Please indicate who or what the source of this experience was. Select all that apply.
   1. Work/employers/coworkers
   2. Immediate family
   3. Extended family
   4. Friends and acquaintances
   5. Members of your church or faith community
   6. Partner/spouse
   7. Healthcare providers, such as physician, nurse, midwife, office staff
   8. Strangers in public places
   9. Media, such as television, news, internet, social media
   10. Other pregnant women or new mothers
   11. Society and social expectations in general
   12. Fill in the blank
   13. This has not happened to me at all.
2. So that we can fully understand what happened, for each of the people or situations you selected above, please provide an example of one of these experiences. Make sure to describe who/what made you feel bad or treated you differently and how it happened.
3. For each of the people or situations you selected above: How often have you been treated differently or been made you feel bad or uncomfortable about your weight since you became pregnant?
   1. Less than once a month
   2. A few times a month
   3. At least once a week
   4. A few times a week
   5. Almost every day
   6. 1 or 2 times a day
   7. 3 or more times a day

**Specific Questions about Healthcare**

The next set of questions will ask you about your experiences in healthcare.

1. How would you describe your overall experiences with prenatal care?
   1. Very negative
   2. Somewhat negative
   3. Neutral
   4. Somewhat positive
   5. Very positive
2. How would you describe your overall experiences with labor and delivery healthcare?
   1. Very negative
   2. Somewhat negative
   3. Neutral
   4. Somewhat positive
   5. Very positive
3. How would you describe your overall experiences with postpartum healthcare?
   1. Very negative
   2. Somewhat negative
   3. Neutral
   4. Somewhat positive
   5. Very positive
4. Have you ever felt or thought any of the following while interacting with pregnancy or post-partum healthcare providers (such as physicians, nurses, etc.) **because of your weight**? Select all that apply.
   1. Judged
   2. Shamed or ashamed
   3. Guilty
   4. Less worthy
   5. Invisible
   6. Unimportant
   7. Disrespected
   8. Negatively compared to other patients
   9. As though the healthcare provider did not like you
   10. As though the healthcare provider thought you were stupid or unintelligent
   11. Valued
   12. Important
   13. Accepted
5. Which healthcare providers made you feel this way? Select all that apply.
   1. Physician
   2. Nurse or Nurse Assistant
   3. Physician’s assistant
   4. Midwife or doula
   5. Ultrasound technician
   6. Office staff
   7. Other – fill in the blank
6. What kind of practice were you in when these experiences occurred? Select all that apply.
   1. Hospital-affiliated OBGYN office
   2. Private practice OBGYN office
   3. Birthing center
   4. Public Clinic
   5. Urgent care
   6. Hospital emergency room or other specialist
   7. Other – fill in the blank
7. At any point during your pregnancy, did you change healthcare providers because of how you were treated related to your weight or weight gain?
   1. Yes
   2. No
8. Which of the following is true of your experiences with healthcare during pregnancy or postpartum?
   1. Too little attention was paid to my weight/weight gain
   2. About the right amount of attention was paid to my weight/weight gain
   3. Too much attention was paid to my weight/weight gain
9. During your pregnancy or in the first year after giving birth, did you ever feel that you could not trust your doctor or that you had to advocate or stand up for yourself because your doctor focused too much on weight?
   1. Yes
   2. No

**Specific Questions about Breastfeeding**

The next set of questions will ask you about breastfeeding.

IF PREGNANT

1. Do you think you will feel uncomfortable seeking help with breastfeeding from a healthcare professional?
   1. Yes
   2. No
2. If yes, is one of the reasons for this due to your weight?

IF POSTPARTUM and IF BREASTFED

1. Have you ever felt uncomfortable seeking help with breastfeeding from a healthcare professional?
   1. Yes
   2. No
2. If yes, is one of the reasons for this due to your weight?
   1. Yes
   2. No
